# Investigating the Poverty-Reducing Effects of SNAP on Non-nutritional Family Outcomes: A Scoping Review

**DOI:** 10.1007/s10995-024-03898-3

**Published:** 2024-02-19

**Authors:** R. William Evans, Zane P. Maguet, Gray M. Stratford, Allison M. Biggs, Michael C. Goates, M. Lelinneth B. Novilla, Megan E. Frost, Michael D. Barnes

**Affiliations:** 1 Department of Public Health, Brigham Young University, Provo, USA; 2 Science Librarians, Harold B. Lee Library, Provo, USA

**Keywords:** SNAP, Family functioning, Family stress model, Policy, Supplemental nutrition assistance program, Poverty, Welfare

## Abstract

**Introduction/Purpose:**

Poverty-reduction efforts that seek to support households with children and enable healthy family functioning are vital to produce positive economic, health, developmental, and upward mobility outcomes. The Supplemental Nutrition Assistance Program (SNAP) is an effective poverty-reduction policy for individuals and families. This study investigated the non-nutritional effects that families experience when receiving SNAP benefits.

**Methods:**

We conducted a scoping review using the PRISMA Guidelines and strategic search terms across seven databases from 01 January 2008 to 01 February 2023 (n=2456). Data extraction involved two researchers performing title-abstract reviews. Full-text articles were assessed for eligibility (n=103). Forty articles were included for data retrieval.

**Results:**

SNAP positively impacts family health across the five categories of the Family Stress Model (Healthcare utilization for children and parents, Familial allocation of resources, Impact on child development and behavior, Mental health, and Abuse or neglect).

**Discussion/Conclusion:**

SNAP is a highly effective program with growing evidence that it positively impacts family health and alleviates poverty. Four priority policy actions are discussed to overcome the unintentional barriers for SNAP: distributing benefits more than once a month; increasing SNAP benefits for recipients; softening the abrupt end of benefits when wages increase; and coordinating SNAP eligibility and enrollment with other programs.

## Significance

**What is already known on this subject?:**

Poverty-reduction efforts that invest in children have especially significant positive benefits by producing positive economic, health, developmental, and upward mobility outcomes. SNAP is among the leading poverty-reducing policies and enrolls the largest number of participants for both nutritional and non-nutritional benefits.

**What this study adds?:**

To our knowledge, no study has synthesized the non-nutritional impact of SNAP on family health. We found that SNAP positively impacts family health across five categories of the Family Stress Model (Healthcare utilization for children and parents, Familial allocation of resources, Impact on child development and behavior, Mental health, and Abuse or neglect). Further, we present four policy actions resulting from this scoping review that deserve attention from policymakers, program administrators, and retailer establishments: distribute benefits more than once a month; increase SNAP benefits for recipients; soften the abrupt end of benefits when wages increase; and coordinate SNAP eligibility and enrollment with other programs.

## Introduction

The Supplemental Nutrition Assistance Program (SNAP) is the largest federal nutrition assistance program in the United States (Caswell & Yaktine, 2013). SNAP provides monthly cash assistance to families meeting eligibility standards through an Electronic Benefit Transfer (EBT) card, which is used to purchase food items at participating grocery stores. The program was launched in 1964 with the passage of the Food Stamp Act. While the overall structure of the program has remained relatively constant, changes to the program (primarily achieved through the periodical reauthorization of the Farm Bill) have included adjustments to its funding amount, modifications to eligibility requirements, and the introduction of an electronic benefit transfer card, among others.

Families with children make up the largest demographic of SNAP participants (Center on Budget & Policy Priorities, 2023). Families with children also make up the largest demographic in poverty (Cellini et al., 2008). Children living below the federal poverty line are associated with increased odds of being overweight (Gupta et al., 2007), worsened Behavioral Problem Index scores (a measure of socio-emotional development) (Lee & Zhang, 2022), and increased odds of poverty later in life that contribute to generational cycles of deprivation (Wagmiller & Adelman, 2009). Poverty-reduction efforts that invest in children have significant benefits and enable healthy family functioning by producing positive economic, health, developmental, and upward mobility outcomes (Collyer et al., 2022).

Given SNAP’s objective to reduce poverty and increase resources for the purchase of food, extensive research has focused on evaluating the nutritional benefits and economic impact of SNAP. (Engel & Ruder, 2020; Holley & Mason, 2019; Mande & Flaherty, 2023; Ryan-Ibarra et al., 2020) For example, studies have monitored the impact of SNAP on the consumption of sugar sweetened beverages (Andreyeva et al., 2015), fruit and vegetable consumption (Verghese et al., 2019), and child weight status (Hudak & Racine, 2019).

While there is much literature and many systematic reviews studying the nutritional benefits of SNAP, an emergent and growing body of literature investigates the impact of SNAP on the family beyond its direct effects on food security and nutrition (Breck, 2018; Engle & Black, 2008; Heflin et al., 2017; Hoynes et al., 2016; Maguire-Jack et al., 2022; Parolin, 2021; Sonik, 2016). A broad review of these non-nutritional impacts of SNAP has not been conducted. Thus, this scoping review focused on the non-nutritional influence of SNAP on family functioning and child health and well-being and their policy implications, including identifying additional areas for research.

## Methods

Following PRISMA guidelines (Page et al., 2021), we conducted a scoping review to explore the non-nutritional impact of SNAP on the family and child health and well-being. Our search terms (see Appendix 1) were informed by the component descriptors of the Family Health Scale (long form) and the Family Stress Model (Crandall et al., 2020). We conducted a search in seven databases (see Appendix 1) using a list of familial terms, outcome terms, and SNAP synonyms on February 1, 2023. We included all articles published from January 1, 2008 to February 1, 2023 given that the 2008 Farm Bill significantly altered governmental nutrition programs, increased funding, and renamed it the Supplemental Nutrition Assistance Program (SNAP). Articles were compiled in EndNote Web and were manually deduplicated.

The codebook (see Appendix 2) and search terms were developed by co-authors, including two reference librarians. Based on the codebook, two co-authors screened the retrieved articles’ titles and abstracts, and the reasons for exclusion were recorded. Questions on the eligibility of articles were resolved through discussions with a third researcher. The articles included in the scoping review were categorized according to Conger et al., (1994) Family Stress Model descriptors. We defined those five family stress outcomes in this study as familial allocation of time/money, mental health of children or parents, abuse or neglect of children or other family members, healthcare utilization for children or parents, and developmental/behavioral results in children. Two co-authors who independently reviewed the articles using the codebook in Google Forms extracted the full-text review data. The entire research team used an objective definition-based consensus discussion to arbitrate less than five eligibility and coding disagreements. Lastly, two researchers reviewed the final number of included articles to compile relevant information into tables. Another trained researcher verified a randomly-selected one-third of the articles.

The inclusion criteria for the scoping review included the following:

- Publication date: 01/01/2008–02/01/2023
- Publication language: English
- Geographic focus: United States
- Study design: Empirical studies only
- Target population: Families/children
- Independent variable: SNAP (participation, eligibility, policies)
- Dependent variables: Non-food-related outcomes

One of the co-authors evaluated the quality of final articles included in this scoping review using the 2018 Mixed Methods Appraisal Tool (MMAT) (Hong et al., 2018) (see Table 2 for the MMAT score for each article). The MMAT appraises the quality of empirical research included in systematic or scoping reviews of mixed studies, allowing different methodological research to be compared to each other. Articles are scored from 1–5, where 1 is the lowest research quality and 5 indicates the highest research quality.

## Results

### Study Characteristics

**Fig. 1:**
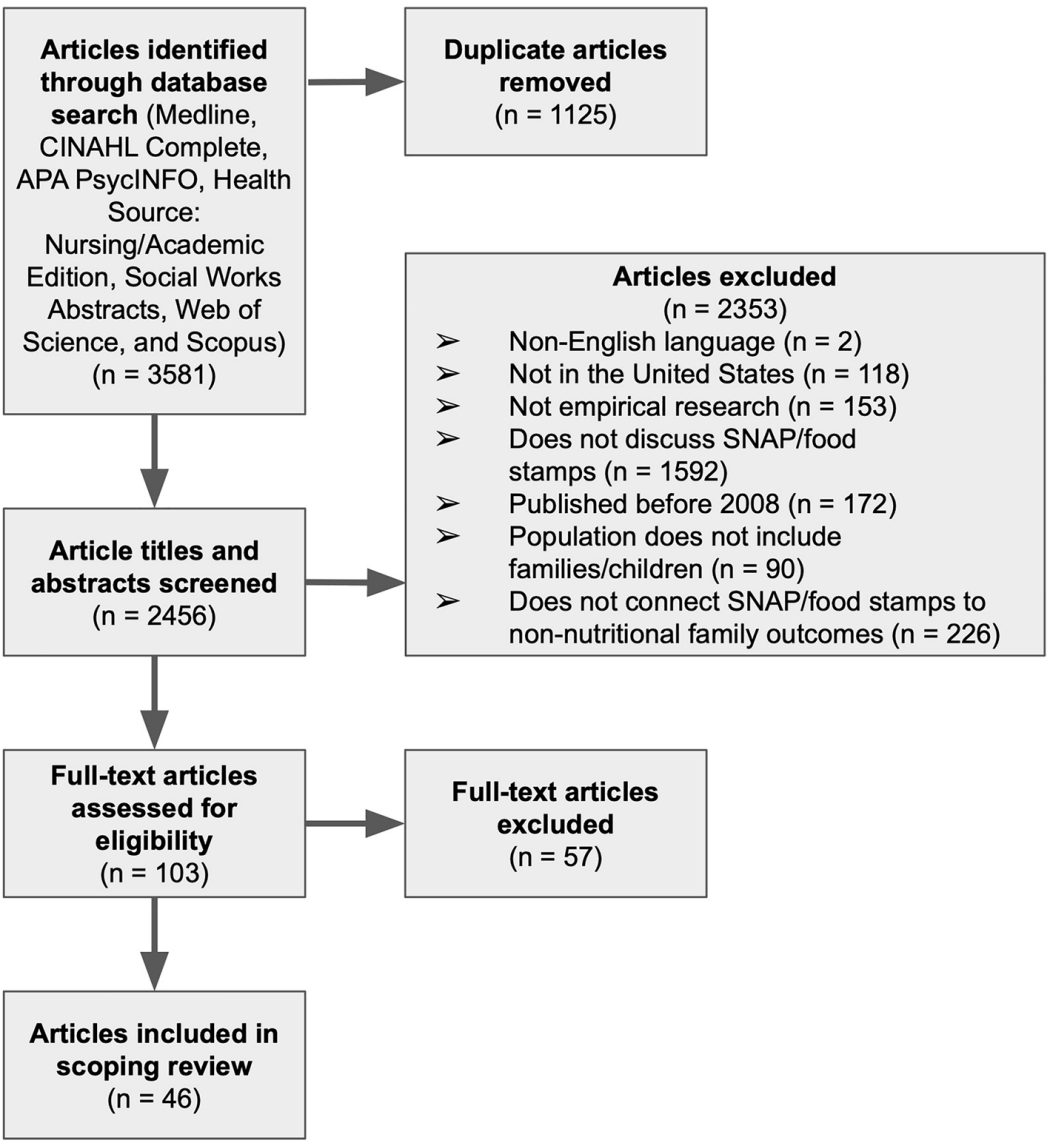
PRISMA diagram

The initial database searches yielded 3581 articles. After deduplication, 1125 articles were excluded, resulting in 2456 articles. Following the title-abstract screening, 2353 articles were excluded, reducing the total to 103 articles for the full-text review. After the full-text review, 57 articles were excluded, resulting in the final total of 46 articles. See Fig. 1 for the PRISMA diagram.

Several different SNAP-related independent variables emerged as ways to examine the impact of this program. The majority of studies compared SNAP participants to socioeconomically comparable, income-eligible SNAP non-participants (n = 27). Other common SNAP-related independent variables included the timing of SNAP fund distribution throughout the month (n = 9) and SNAP participation before and after a policy or implementation change (n = 4). The methodological design of included studies were quantitative (n = 43), qualitative (n = 2), and mixed-methods (n = 1). See Table 2 for a full listing of SNAP-related independent variables and study designs. Major themes identified included SNAP’s effect on healthcare utilization for children or parents (n = 9), familial allocation of resources such as time or money (n = 15), behavioral or developmental results in children (n = 12), mental health of children or parents (n = 7), and abuse or neglect of children or other family members (n = 8). The majority of articles (n = 27) had the highest MMAT score of 5, indicating high empirical research quality. Fewer articles (n = 16) had an MMAT score of 4, while only a few (n = 3) articles had an MMAT score of 3. Of studies with an MMAT score of 3, two studies were categorized under familial allocation of resources, and one was grouped with articles on mental health. No articles were excluded based on MMAT findings. See Table 1 for a description of included studies. The five major themes are expounded upon below.

**Table 1:** Description of included studies (n = 46)

Citation	Article title	Geographic location(s)	Year data was collected	Participants involved	Racial/ethnic composition	Methods	Measure(s)/data source(s)
Almada & McCarthy, (2017)	It’s a cruel summer: Household responses to reductions in government nutrition assistance	National	1996–2014	Whole family	80.22% White, 12.33% Black, 7.45% Hispanic	Quantitative	Consumer Expenditure Survey
Arteaga et al., (2021)	Giving kids a boost: The positive relationship between frequency of SNAP participation and Infant’s preventative health care utilization	Missouri	2006–2014	Children	67.7% non-Hispanic White, 26.3% non-Hispanic Black, 2.2% Hispanic, 3.9% multiple races, other race, or unknown/missing	Quantitative	Missouri Department of Social Services, Family Support Division
Austin et al., (2023)	Association of State Expansion of Supplemental Nutrition Assistance Program Eligibility With Rates of Child Protective Services-Investigated Reports	National	2006–2019	Whole family	CPS-investigated reports: 19.8% Black children, 45.7% White children	Quantitative	National Child Abuse and Neglect Data System (NCANDS) Child Files
Barr & Smith, (2022)	Fighting Crime in the Cradle: The Effects of Early Childhood Access to Nutritional Assistance	North Carolina	1960s, 1972–2015	Children	Not reported	Quantitative	North Carolina criminal activity records, FBI criminal activity records, North Carolina food stamp program rollout by county
Beatty et al., (2013)	Time to eat? The relationship between food security and food-related time use	National	2004–2010	Whole family	Single: 77.3% White, 21.8% Black, 0.2% American Indian, 0.0% Asian Married: 91.0% White, 7.5% Black, 0.1% American Indian, 0.2% Asian	Quantitative	Current Population Survey-Food Security Supplement (FSS) and the American Time Use Survey (ATUS)
Bensignor et al., (2021)	The Relationship between Household SNAP Participation, Parent Feeding Styles, and Child Eating Behaviors	Minneapolis-Saint Paul, Minnesota	2012–2017	Children and mothers	17.8% non-Hispanic White, 19.3% non-Hispanic Black, 54.5% Hispanic, 4.1% multiracial, 4.3% other	Quantitative	Minnesota Now Everybody Together for Amazing Healthful Kids (NET- Works) Child Eating Behavior Questionnaire (CEBQ), Parental Feeding Styles Questionnaire
Bergmans et al., (2017)	Participation in the Supplemental Nutrition Assistance Program and maternal depressive symptoms: Moderation by program perception	National	1998–2005	Mothers	62.1% non-Hispanic Black, 21.5% Hispanic, 13.3% non-Hispanic White, 3.1% other	Quantitative	Composite International Diagnostic Interview Short Form (CIDI–SF) in the Fragile Families and Child Wellbeing Study (FFCWS)
Bolbocean & Tylavsky, (2021)	The impact of safety net programs on early-life developmental outcomes	Memphis, Shelby County, Tennessee	2006–2011	Children	66% African American, 31% Caucasian	Quantitative	The CANDLE Study. Bayley Scales of Infant Development-III (BSID-III) scores, cognitive and language outcomes
Bronchetti et al., (2019)	Local food prices, SNAP purchasing power, and child health	National	1999–2010	Children	16% Black, 19% Hispanic	Quantitative	Regional cost of the Thrifty Food Plan (TFP), National Health Interview Survey (NHIS), December Current Population Survey (CPS)
Bullinger et al., (2021)	Proximity to SNAP-authorized retailers and child maltreatment reports	Connecticut	2011–2015	Whole family	Racial/ethnic composition for defined geographical blocks, which are 100% urban, 99% urban, 80% urban, 23% urban, 100% urban, 100% urban, respectively: White: 25.2%, 50.5%, 64.8%, 76.0%, 84.3%, 89.9% Black: 27.4%, 16.1%, 11.3%, 6.6%, 3.2%, 1.6% Hispanic: 41.0%, 26.0%, 16.9%, 10.6%, 6.2%, 3.7%	Quantitative	Child Protective Services reports
Carr & Packham, (2020)	SNAP Schedules and Domestic Violence	Chicago, Illinois	2009–2011	Children and mothers	Not reported	Quantitative	Chicago crime records, City of Chicago’s online data portal for crimes
Cheng, (2010)	Financial self-sufficiency or return to welfare? A longitudinal study of mothers among the working poor	National	1995–2000	Mothers	43.4% White, 40% African American, 16.6% Hispanic	Quantitative	Survey of Income and Program Participation (SIPP)
Cho & Lightfoot, (2022)	Recurrence of Substantiated Maltreatment Reports between Low-Income Parents With Disabilities and Their Propensity-Score Matched Sample Without Disabilities	5 unspecified sites	1991–2012	Whole family	Parents with disabilities: 37.8% White, 48% Black, 14.2% other Parents without disabilities: 31.5% White, 54.5% Black, 14% other	Quantitative	Longitudinal Studies of Child Abuse and Neglect (LONGSCAN) dataset
Cotti et al., (2020)	Hunger pains? SNAP timing and emergency room visits	South Carolina	2000–2012	Whole family	51% Black, 35% White	Quantitative	SNAP records and Medicaid records
Cotti et al., (2018)	When does it count? The timing of food stamp receipt and educational performance	South Carolina	2000–2012	Children	65% Black	Quantitative	South Carolina Dept of Education, Pre-Admission Content test and the Assesment of State Standards Test
East, (2020)	The Effect of Food Stamps on Children’s Health: Evidence from Immigrants’ Changing Eligibility	National	1996–2015	Children	Children of immigrants: 50% White, 6% Black, 80% Hispanic, 3% Asian Children of natives: 72% White, 22% Black, 11% Hispanc, 0% Asian	Quantitative	National Health Interview Survey (NHIS)
Ettinger de Cuba et al., (2019a)	SNAP, Young Children’s Health, and Family Food Security and Healthcare Access	Boston, Massachusetts; Baltimore, Maryland; Minneapolis, Minnesota; Little Rock, Arkansas; and Philadelphia, Pennsylvania	2006–2016	Whole family	28.0% Latinx, 13.3% White, non-Latinx, 55.4% Black, non-Latinx, 3.3% other	Quantitative	Children’s HealthWatch. National Health and Nutrition Examination Survey (NHANES), parents’ Evaluation of Developmental Status
Ettinger de Cuba et al., (2019b)	Loss of SNAP is associated with food insecurity and poor health in working families with young children	Boston, Massachusetts; Baltimore, Maryland; Minneapolis, Minnesota; Little Rock, Arkansas; and Philadelphia, Pennsylvania	2007–2015	Whole family	30.1% Hispanic, 13.0% non-Hispanic White, 53.6% non-Hispanic Black, 3.2% non-Hispanic other	Quantitative	Children’s HealthWatch
Frank & Sato, (2022)	Food Insecurity and Depressive Symptoms Among Adolescents: Does Federal Nutrition Assistance Act as a Buffer?	Unspecified Midwestern state	2022	Children	Race: 68.1% White, 22.7% Black, 0.7% Asian. 8.5% Other/more than one Ethnicity: 2.8% Hispanic or Latinx, 89.4% not Hispanic or Latinx, 7.8% other	Quantitative	Individual survey. Food insecurity: Child Food Security Survey Module Depressive Symptoms: Reynolds Adolescent Depression Scale
Gassman-Pines & Bellows, (2018)	Food instability and Academic Achievement: A Quasi-Experiment Using SNAP Benefit Timing	North Carolina	2011–2012	Children	47% Black, 10% Hispanic, 35% White, 8% other	Quantitative	EOG (End-of-Grade) school tests
Gennetian et al., (2016)	Supplemental Nutrition Assistance Program (SNAP) Benefit Cycles and Student Disciplinary Infractions	Chicago, Illinois	2005–2006	Children	50.9% Black, 37.5% Hispanic, 11.6% White/other	Quantitative	Chicago Public Schools System enrollment and discipline data, CPS data on disciplinary incidents
Hong et al., (2020)	Are Children of Welfare Recipients at a Heightened Risk of Bullying and Peer Victimization?	National	2003, 2007, 2011–2012, 2016	Children	7.75% two or more races, 76.26% non-Hispanic White, 6.48% Black or African American, 0.81% American Indian/Alaskan, 5.74% Asian, 0.32% Native Hawaiian/Other Pacific, 2.64% some other race	Quantitative	National Survey of Children’s Health (NSCH)
Hong & Henly, (2020)	Supplemental nutrition assistance program and school readiness skills	National	2001–2008	Children	31% Black, 36% Hispanic, 27% White, 6% other	Quantitative	Early Childhood Longitudinal Study-Birth Cohort (ECLS-B)
Johnson-Motoyama et al., (2022)	Association Between State Supplemental Nutrition Assistance Program Policies, Child Protective Services Involvement, and Foster Care in the US, 2004–2016	National	2004–2016	Children	Not reported	Quantitative	SNAP Policy Database22 and the SNAP State Options Reports, the National Child Abuse and Neglect Data System (NCANDS) Child File and the Adoption and Foster Care Reporting and Analysis System (AFCARS)
Kinsey et al., (2019)	Food and financial coping strategies during the monthly Supplemental Nutrition Assistance Program cycle	Philadelphia, Pennsylvania	2016–2017	Mothers	100% Black	Mixed Methods	Qualitative interviews and surveys
Laurito & Schwartz, (2019)	Does School Lunch Fill the “SNAP Gap”at the End of the Month?	National	2012–2013	Children	SNAP: 71.61% non-White Non-SNAP: 54.14% non-White	Quantitative	NSLP participation via FoodAPS
Mabli & Worthington, (2017)	Supplemental Nutrition Assistance Program Participation and Emergency Food Pantry Use	National	2011–2012	Whole family	26.5% non-Hispanic Black, 48.0% non-Hispanic White, 7.0% non-Hispanic other, 22.5% Hispanic	Quantitative	SNAP Food Security survey
McKernan et al., (2021)	The effect of the US safety net on material hardship over two decades	National	1992–2011	Whole family	16.1% non-Hispanic Black, 13.6% Hispanic, 5.3% other non-Hispanic & non-White	Quantitative	Survey of Income and Program Participation (SIPP)
Miller & Morrissey, (2021)	SNAP participation and the health and health care utilisation of low-income adults and children	National	2008–2013	Whole family	26.6–44.2% White, not Hispanic; 14.5–27.6% Black, not Hispanic; 2.4–7.2% Asian, not Hispanic; 29.1–51.2% Hispanic, any race; 1.1–2.4% other	Quantitative	NHIS
Millett et al., (2011)	Are economic trends associated with child maltreatment? Preliminary results from the recent recession using state level data	Arizona, California, Massachusetts, Missouri, North Carolina, Oregon, and Wisconsin	2000–2010	Whole family	Not reported	Quantitative	CPS reports
Morris et al., (2019)	County-level socioeconomic and crime risk factors for substantiated child abuse and neglect	Tennessee	2004–2016	Children	Not reported	Quantitative	KIDS COUNT Data Center, Tennessee Bureau of Investigation’s Incident Based Reporting System
Morrissey & Miller, (2020)	Supplemental Nutrition Assistance Program Participation Improves Children’s Health Care Use: An Analysis of the American Recovery and Reinvestment Act’s Natural Experiment	National	2008–2010	Children	Not reported	Quantitative	NHIS
Munger et al., (2016)	The Role of the Supplemental Nutrition Assistance Program in the Relationship Between Food Insecurity and Probability of Maternal Depression	National	Not reported	Children and mothers	42% non-Hispanic Black, 30% Hispanic, 24% non-Hispanic White, 4% other	Quantitative	Major Depression Episode subscale of the Composite International Diagnostic Interview–Short Form, Fragile Families and Child Wellbeing Study
Nieves et al., (2022)	“Come with us for a week, for a month, and see how much food lasts for you:” A Qualitative Exploration of Food Insecurity in East Harlem, New York City	East Harlem, New York City, New York	2018	Parents	45.9% Latinx, 24.3% Asian, 21.6% Black, 2.7% White, 2.7% Multiracial, 2.7% other	Qualitative	Individuals surveys and interviews
Parolin, (2021)	Income Support Policies and the Rise of Student and Family Homelessness	National	2014–2018	Children	Not reported	Quantitative	Department of Education
Powell et al., (2018)	Predicting household residency among youth from vulnerable families	Baltimore, Maryland	2005–2007	Children and mothers	95% Black	Quantitative	Audio-Computer Assisted Self-Interviewing
Pryor et al., (2023)	Childhood food insecurity, mental distress in young adulthood and the supplemental nutrition assistance program	National	2005–2013	Children, Whole family	Not reported	Quantitative	Kessler 6-item Non-Specific Distress Scale for psychological distress, Composite International Diagnostic Interview 2-item clinical depression screener
Rogers et al., (2022)	Supplemental Nutrition Assistance Program participation and health care expenditures in children	National	2013–2017	Children	SNAP: 33.2% Hispanic, 29.4% non-Hispanic Black, 26.9% non-Hispanic White, 9.6% other Non-SNAP: 40.1% Hispanic, 14.0% non-Hispanic Black, 33.8% non-Hispanic White, 11.3% other,	Quantitative	Medical Expenditure Panel Survey (MEPS)
Rothbart & Heflin, (2023)	Inequality in literacy skills at kindergarten entry at the intersections of social programs and race	Virginia	2014–2017	Children	50.6% White, 23.7% Black, 14.5% Hispanic, 4.8% Asian/Pacific islander, 6.4% multiracial or other	Quantitative	Virginia Departments of Education (VDOE) and the Department of Social Services (VDSS) administrative data, PALS
Schenck-Fontaine et al., (2017)	Use of informal safety nets during the supplemental nutrition assistance program benefit cycle: How poor families cope within-month economic instability	Durham, North Carolina	2015	Whole family	87% African American, 5.4% White, 5.7% Hispanic, and 1.5% other	Quantitative	Survey of a sample of SNAP households with children
Shaefer & Gutierrez, (2013)	The Supplemental Nutrition Assistance Program and Material Hardships among Low Income Households with Children	National	1996, 2001, and 2004 panels (3–4 years each)	Whole family	60% White, 25% Black, 15% Hispanic	Quantitative	US Census Bureau Survey of Income and Program Participation
Steimle et al., (2021)	Understanding patterns of food insecurity and family well-being amid the COVID-19 pandemic using daily surveys	Rural Pennsylvania	2020	Whole family	61% Latinx, 16% White, 9% Black, 14% mixed/other race	Quantitative	Individual text message survey
Vartanian et al., (2011)	Food stamps and dependency: Disentangling the short-term and long-term economic effects of food stamp receipt and low income for young mothers	National	1968–2005	Mothers	53% White, 42% Black, 4% neither	Quantitative	PSID survey
Wang et al., (2020)	The effects of welfare participation on parenting stress and parental engagement using an instrumental variables approach: Evidence from the Supplemental Nutrition Assistance Program	National	1996, 2001, 2004, 2008	Whole family	SNAP: 34.35% non-Hispanic White, 33.20% non-Hispanic Black, 24.38% Hispanic, 1.59% Asian, 6.47% other Non-SNAP: 65.98% non-Hispanic White, 11.33% non-Hispanic Black, 15.55% Hispanic, 3.64% Asian, 3.51% other	Quantitative	Survey of Income and Program Participation, temporal and state variations in SNAP policy rules
Weinstein et al., (2018)	What Works When It Comes to Having Enough: A Qualitative Analysis of SNAP-Participants’ Food Acquisition Strategies	Lawrence, Fall River, and Holyoke, Massachusetts	2017–2018	Parents	47% non-Hispanic White, 24% Dominican, 19% Puerto Rican, 10% other	Qualitative	Focus groups
Xu et al., (2021)	Material hardship and child neglect risk amidst COVID-19 in grandparent-headed kinship families: The role of financial assistance	National	2020	Grandparent headed households	68% White, 9.5% Black, 20% Hispanic, 2% other	Quantitative	The conflict tactics scales parent–child

### Healthcare Utilization for Children and Parents

The majority of examined studies (n = 7) found a positive association between SNAP participation and receiving needed medical care (Arteaga et al., 2021; Bronchetti et al., 2019; Ettinger de Cuba et al., 2019a, 2019b; Miller & Morrissey, 2021; Morrissey & Miller, 2020; Shaefer & Gutierrez, 2013). In order to control for income, most studies limited their study population to either those under a certain income threshold, who did not have private health insurance, or who participated in SNAP (n = 7). One study looked at ecological data, and another controlled for income through statistical analysis. Those who became SNAP ineligible due to income increases experienced more missed healthcare. Results also showed that higher numbers of early childhood wellness visits were correlated to increases in SNAP benefit amounts and the purchasing power of SNAP benefits relative to the local cost of food items. One study found that SNAP participants were more likely than their non-SNAP counterparts to receive needed dental care and eyeglasses. However, they were also more likely to delay seeking care and could not afford prescription medication (Miller & Morrissey, 2021). Another study found no major difference in healthcare expenditures for children between SNAP participants and non-SNAP participants (Rogers et al., 2022). Regarding the timing of benefit distribution, it was found that non-urgent emergency room visits were lower on the day of SNAP benefit disbursement than on other days for all ages (Cotti et al., 2020).

A variety of methods were used to account for the potentially confounding role of public health insurance programs such as Medicaid in these results. Some study samples (Arteaga et al., 2021; Cotti et al., 2020) only included Medicaid participants, while other studies (Bronchetti et al., 2019; Miller & Morrissey, 2021; Morrissey & Miller, 2020) specifically controlled for Medicaid or other health insurance. The remaining studies that found a correlation between SNAP participation and healthcare usage (Ettinger de Cuba et al., 2019a, 2019b; Shaefer & Gutierrez, 2013) used various proxy measures to ensure that the entire sample was low-income and therefore did not vary with regard to Medicaid eligibility.

### Familial Allocation of Resources

SNAP participation impacted resource management in various ways, and a majority of articles indicated improved resource allocation (n = 8). Most studies controlled for the effects of income by limiting their study population below a certain percentage of the federal poverty line (n = 6) or to only SNAP participants (n = 6); those who didn’t looked at other measures, such as food security or youth homelessness (n = 3). SNAP participation reduced housing and utility payment delays (Shaefer & Gutierrez, 2013) and overall material hardship (McKernan et al., 2021), and reducing SNAP benefits led to increased odds of housing instability and being behind on utility bills (Ettinger de Cuba et al., 2019b). Several studies (n = 5) noted that SNAP participants experience a majority of the poverty-reducing effects of SNAP towards the beginning of the month, and rely more on non-SNAP assistance, such as food pantries or social networks, at the end of the benefit cycle than at the beginning (Kinsey et al., 2019; Laurito & Schwartz, 2019; Nieves et al., 2022; Schenck-Fontaine et al., 2017; Weinstein et al., 2018). The literature is inconclusive as to SNAP’s effects on family homelessness (Parolin, 2021) as well as young mothers’ usage of other public assistance (Cheng, 2010; Mabli & Worthington, 2017; Vartanian et al., 2011). SNAP was associated with less time spent on meal preparation, non-grocery food shopping (i.e., prepared food, fast food, etc.), and eating (Beatty et al., 2013). For a full list of results, see Table 2.

**Table 2:** Key takeaways

Citation	Independent variable	Dependent variable	MMAT score	Summary of findings	Policy implications
Healthcare utilization for children or parents					
Arteaga et al., (2021)	SNAP participation level (always on SNAP. leaving SNAP, receiving SNAP unstably)	Preventative infant healthcare (well visits and vaccination rates)	5	Stable SNAP participation for 10 + months increases the likelihood of a child receiving 5 + well-child visits, especially for Hispanics, Blacks, and infants living in urban areas	How SNAP’s recertification process may be simplified to reduce barriers to consistent SNAP enrollment
Bronchetti et al., (2019)	Differences across State policy/implementation of variation in SNAP purchasing power	Children’s health care utilization and health	4	Increases in SNAP purchasing power increase the likelihood of a child receiving a well child visit and missing 1 less day of school due to illness, but do not signficiantly impact health status	Adjusting SNAP benefit levels to be comensurate with local food price variations to reduce healthcare utilization and absenteeism
Cotti et al., (2020)	Early in the SNAP benefit cycle versus late in the SNAP benefit cycle (i.e. changes throughout the SNAP month)	ER visits for all ages	5	Non-urgent ER visits are lower on the day of SNAP receipt than on other days for all ages	Spreading SNAP benefit distribution throughout the month may improve health outcomes for older adults (55 +) who utilize the ER more at the end of the SNAP benefit cycle
Ettinger de Cuba et al., (2019a)	SNAP participation versus no SNAP participation	Developmental risk and healthcare cost sacrifices	5	The adjusted odds of developmental risk and health care cost sacrifices were lower among SNAP participants than among nonparticipants	None recorded
Ettinger de Cuba et al., (2019b)	Various levels of SNAP participation (e.g. consistently on SNAP, sometimes on SNAP, etc.) from changes in SNAP eligibility due to increased income or reduced benefits	Foregone healthcare	5	Reduced SNAP benefits from income increases resulted in more forgone healthcare. Those facing SNAP cutoff have increased likelihood of healthcare cost sacrifices	Reduce the abrupt end or reduction of SNAP with increasing wages, and do not implement work requirements for applicants with children. Workers in volatile jobs may have a month-to-month change in SNAP benefits that needs to be addressed
Miller & Morrissey, (2021)	SNAP participation versus no SNAP participation	Healthcare usage in children/parents	5	SNAP increased the probability of very good or excellent health among adults and the probability of needing and receiving dental care or eye glasses. However, children on SNAP were more likely to report behavioral problems, needing but not being able to afford prescription medications, being in fair or poor health, having stomach problems, and having a cold in the last two weeks. Adults on SNAP also were more likely to be in fair or poor health, spend more days in bed, have psychological distress, delay seeking care, and be out of work	Expanding SNAP may improve health and other practical outcomes. More research is needed to understand the mechanisms of how SNAP affects child health
Morrissey & Miller, (2020)	SNAP expansion due to the American Recovery and Reinvestment Act (ARRA) beginning in April 2009	Outstanding health needs among children, Outstanding medication needs among children, Needing but being unable to afford healthcare (children), Likelihood of having seen a general doctor in the past 12 months (children)	4	Increasing SNAP benefits decreased the amount of low-income children who needed but could not afford medical care by 65% compared to low-income, SNAP inelgible children. This association was stronger for single-parent households. Children who received SNAP were also more likely to have seen a doctor in the past year	Small increases in SNAP benefits may reduce disparity in health care access
Rogers et al., (2022)	SNAP participation versus no SNAP participation	Health care expenditures	5	No major difference in health care expenditures between SNAP and non-SNAP participants were identified for children	SNAP should not be evaluated for its effectiveness in reducing health care expenditures but as an investment in improving child well-being
Shaefer & Gutierrez, (2013)	SNAP participation versus no SNAP participation	Foregone hospital visit when needed	5	SNAP reduces nonfood material hardships including reducing needing, but not receiving hospital care	Increasing SNAP benefits would likely decrease food and nonfood material hardship across the US
Familial allocation of resources such as time or money					
Almada & McCarthy, (2017)	SNAP participation before versus after a policy/implementation change (i.e. natural experiment)	Reallocation of household funds in the summer	5	SNAP households significantly increase food expenditures during the summer, significant reduction in entertainment and "other" expenditures	SNAP’s inability to cover meal costs without the aid of National School Lunch program. Inflexibility of household budgets in absorbing benefit reductions
Beatty et al., (2013)	SNAP participation versus no SNAP participation	Time spent on food preparation activities	5	SNAP associated with less time spent in meal preparation, non-grocery food shopping, and eating	Time constraints as the major obstacle for SNAP participant to eat well. Thrifty Food Plan may require too much time for cooking. Save time by cutting out administrative paperwork. Work requirements may crowd out time for meal preparation
Cheng, (2010)	SNAP participation versus no SNAP participation	Odds of becoming TANF welfare dependent again	5	Odds of food stamp working mother to become a TANF recipient again are 1.007 times greater than women not receiving food stamps	Suggests that food stamps are ineffective in lifting working mothers out of poverty, recommends that food stamps and Medicaid should be secondary to "growing human capital."
Ettinger de Cuba et al., (2019b)	Various levels of SNAP partcipation (e.g. consistently on SNAP, sometimes on SNAP, etc.)	Housing/energy payment instability	5	Reduction of SNAP benefits associated with increased odds of housing instability and energy insecurity (behind on bills)	Recommended smaller, incremental reductions in SNAP benefits at a time. Having a longer reporting period for income for those with volatile work conditions
Kinsey et al., (2019)	Early in the SNAP benefit cycle versus late in the SNAP benefit cycle (i.e. changes throughout the SNAP month)	Coping strategies at the end of the month for SNAP mothers	3	Significant energy spent on budgeting, skipped meals, purchasing unhealthier foods, relied on outside support at the end of the month	More frequent, smaller SNAP distributions could ease the cognitive load of making ends meet at the month
Laurito & Schwartz, (2019)	Early in the SNAP benefit cycle versus late in the SNAP benefit cycle (i.e. changes throughout the SNAP month)	Household dependence on school lunches during SNAP benefit cycle	4	SNAP households rely more on school lunch and breakfast towards the end of the SNAP month	Consider interaction between SNAP and school lunches
Mabli & Worthington, (2017)	Various levels of SNAP participation (e.g. consistently on SNAP, sometimes on SNAP, etc.)	Food pantry use	5	Participating in SNAP for 6 months was associated with decreased food pantry use	Consider part-time work as insufficient to meet food needs in SNAP households. Collect more diverse information on how needs are being met in SNAP households
McKernan et al., (2021)	SNAP participation versus no SNAP participation	Number of "material hardships," e.g. inability to pay bills and rent	5	Receving SNAP reduces the number of material hardships	The positive effects of SNAP on the economy
Nieves et al., (2022)	Early in the SNAP benefit cycle versus late in the SNAP benefit cycle (i.e. changes throughout the SNAP month)	Coping with food insecurity at the end of the SNAP month	5	Sharing SNAP benefits, meals, and groceries with family and friends at the end of the SNAP month	Recommends increasing the monthly SNAP allocation
Parolin, (2021)	SNAP accessibility and generosity	Share of all elementary students who experience homelessness	5	No conclusive evidence that greater access to SNAP reduces family homelessness	No specific recommendations for SNAP
Powell et al., (2018)	SNAP participation versus no SNAP participation	Likelihood of not living with biological mother	4	Food stamps were not associated with an increased likelihood that children were not living with their biological mother	Welfare programs should should examine the interdependence and interactions of various kinds of caregivers
Schenck-Fontaine et al., (2017)	Early in the SNAP benefit cycle versus late in the SNAP benefit cycle (i.e. changes throughout the SNAP month)	Coping strategies throughout the SNAP benefit cycle	3	Borrowing money later in month, using a food bank, using up benefits in the first week	More frequent, smaller SNAP distributions. Also, consider the inadequacy of SNAP benefits to last a month
Shaefer & Gutierrez, (2013)	SNAP participation versus no SNAP participation	Difficulty meeting household expenses (utilities, rent, bills)	5	SNAP reduces risk that housholds will fall behind on housing and utility payments	Consider the material hardship benefits that SNAP provides to households
Vartanian et al., (2011)	SNAP participation versus no SNAP participation	Dependence of young mothers on public assistance programs	5	No evidence that young mothers who receive SNAP are more or less dependent on public assistance programs then other low-income non-SNAP young mothers	None recorded
Weinstein et al., (2018)	Early in the SNAP benefit cycle versus late in the SNAP benefit cycle (i.e. changes throughout the SNAP month)	Coping strategies used at the end of the SNAP benefit cycle	5	Adults sacrificing meals for children, utilizing food pantries, borrowing money, put off financial obligations, utilize family networks	Pairing SNAP benefits with time management/strategic living/educational training
Behavioral or developmental results in children					
Barr & Smith, (2022)	SNAP participation versus no SNAP participation	Conviction of a crime	4	For each additional year of food stamp (i.e. SNAP) availability in early childhood, the likelihood of being arrested in young adulthood is reduced by 0.03 arrests per 100 people, or roughly 3%	Reductions in violent crime translate to large external benefits for society. These types of future external benefits are frequently ignored in discussions of the value of social safety net programs. Even under conservative assumptions, the social savings from crime reduction alone outweigh the cost of the program
Bensignor et al., (2021)	SNAP participation versus no SNAP participation	Child eating behaviors	5	No relationship was found between SNAP participation and child eating behaviors (e.g. desire for food, avoidance of food)	None
Bolbocean & Tylavsky, (2021)	SNAP participation versus no SNAP participation	Aggregate development, receptive and expressive communication, and cognitive scores (BSID-III)	4	SNAP has a positive and significant effect on standardized BSID-III scores measuring aggregate development, receptive and expressive communication, as well as cognitive scores	Presented empirical evidence might be critical at a time when funding for SNAP or other safety-net programs is in peril
Cotti et al., (2018)	Early in the SNAP benefit cycle versus late in the SNAP benefit cycle (i.e. changes throughout the SNAP month)	Students’ performance in math exams	4	There is a statistically significant negative relationship between particularly long gaps between SNAP receipt and school test dates. Further, the receipt of benefits on the 4 days prior to the exam is also associated with significantly worse scores	Possibilities to smooth consumption include splitting the benefit distribution into multiple days in a month or using other interventions or adjustments in benefits
East, (2020)	SNAP participation versus no SNAP participation	Developmental health index score in children	5	An additional year of parental food stamp (i.e. SNAP) access results in a decrease in the developmental health index for children of 0.08 standard deviations, which indicates a lack of hindrances to child development	Understanding the effects of previous restrictions in immigrants’ SNAP access is crucially important. The loss of parental Food Stamp eligibility has a large effect on contemporaneous Food Stamp receipt, and loss of parental eligibility before age 5 negatively affects children’s health in the medium-run
Ettinger de Cuba et al., (2019a)	SNAP participation versus no SNAP participation	Developmental risk	5	The adjusted odds of developmental risk were lower among SNAP participants than among nonparticipants	None
Ettinger de Cuba et al., (2019b)	Various levels of SNAP participation (e.g. consistently on SNAP, sometimes on SNAP, etc.)	Developmental risk	5	Developmental risk was marginally associated with reduced SNAP benefits compared to households with consistent participation	SNAP eligibility based on monthly reporting of work activities without regard to the stability of employment may result in families’ losing eligibility for other critical supports that are tied to SNAP. Averaging income over a longer period of time could provide a more realistic picture of family employment and contribute to a more effective “off-ramp” from SNAP
Gassman-Pines & Bellows, (2018)	Early in the SNAP benefit cycle versus late in the SNAP benefit cycle (i.e. changes throughout the SNAP month)	EOG math and reading test scores	4	Relative to nonrecipient students, SNAP-recipient students scored lower on End-of-Grade exams. Controlling for race/ethnicity, gender, and grade, as well as school fixed effects, the difference between SNAP-recipient and nonrecipient students was 0.36 standard deviations for math and 0.35 standard deviations for reading	Although SNAP is an important support, benefits may be insufficient for many families. Findings suggest that increasing benefit amounts would have the benefit of improving school achievement outcomes for low-income children
Gennetian et al., (2016)	Early in the SNAP benefit cycle versus late in the SNAP benefit cycle (i.e. changes throughout the SNAP month)	School disciplinary infractions	5	Controlling for student and school characteristics, estimates show that student disciplinary infractions generally spike at the end of the month irrespective of SNAP receipt status. However, spikes are exacerbated among students who receive SNAP benefits and are further removed from a benefits transfer. The within-month difference in disciplinary infractions for students in SNAP recipient families is 7 percentage points larger than for non recipients. These differences are particularly pronounced for males	Students could substantially benefit from increases to SNAP benefits as a strategy to reduce hunger that may be contributing to increased disciplinary infractions. Other options are altering the timing and frequency of disbursement, changing education policy and practice, and making food available for students during times of income scarcity
Hong et al., (2020)	SNAP participation versus no SNAP participation	Child bully perpetration and peer victimization	5	Bullying victimization and perpetration were positively associated with SNAP	SNAP carries a stigma that children experience at school that makes them a target. Programs that could reduce stigma to protect children from bullying victimization, such as universal free meals for students, should be considered
Hong & Henly, (2020)	SNAP participation versus no SNAP participation	School readiness skills–specifically early math, early reading, and approaches to learning	4	Among students experiencing deep poverty, SNAP participation was found to aid development of early mathatical skills. However, no effect was found on early reading skills	Results provide strong support for the role of SNAP in advancing key school readiness skills that are important to children’s developmental outcomes. SNAP’s impact is comparable in size to that of other key public benefits
Rothbart & Heflin, (2023)	SNAP participation versus no SNAP participation	School performance	4	Literacy and phonological awareness skills were greatest among children that did not participate in any of the three social programs considered, followed by those who are only eligible for free/reduced-price school meals, and then those who participate in SNAP, and finally those who participate in TANF. Race modifies the relationship between program participation and Phonological Awareness Literacy Screening scores; Black and Asian children who participate in public supports typically outperform similar program-participating White children, with Hispanic children lagging further behind	Greater financial and educational supports for those who already participate in SNAP might be warranted, particularly in early childhood
Mental health of children or parents					
Bergmans et al., (2017)	SNAP participation versus no SNAP participation	Depression (maternal), measured using a questionnaire	3	SNAP was associated with a lower risk of depression only if SNAP users percieved themselves as maintaining personal freedom	Suggests continued integration of services with SNAP, such as health insurance, to decrease stigma and negative perceptions
Ettinger de Cuba et al., (2019b)	Various levels of SNAP participation (e.g. consistently on SNAP, sometimes on SNAP, etc.)	Maternal depressive symptoms	5	Reductions in SNAP were correlated with a higher likelihood of maternal depressive symptoms	Cutting off SNAP suddenly is likely to negatively impact health, and when looking at policy policymakers should be aware of churning and other administrative hurdles. The study also suggests making SNAP benefit loss more of a smooth ‘off-ramp’ to avoid making families who increase their income above the limit worse off than before
Frank & Sato, (2022)	SNAP participation versus no SNAP participation	Buffering of food insecurity’s impact on adolescent depressive symptoms	5	Results of this preliminary study suggest that SNAP can alleviate adolescent depression in the context of food insecurity	None
Munger et al., (2016)	SNAP participation versus no SNAP participation	Maternal depression	4	Gaining SNAP was associated with a decrease in the probability of depression; losing SNAP was associated with an increase	SNAP and other safety net programs are very important in ensuring child welfare, but they need a sufficient size and scope
Pryor et al., (2023)	SNAP participation versus no SNAP participation	Attenuation of psychological distress in young adulthood associated with childhood household food insecurit and young adults’ depression	5	When dealing with persistent food insecurity during childhood, SNAP decreased the odds of mental illness in later young adulthood	Increasing access to SNAP for families may decrease the negative effects of food insecurity on later mental health
Steimle et al., (2021)	SNAP participation versus no SNAP participation	Parent psychological distress, child psychological distress	4	SNAP helped parental mood/stress, if the family could use their benefits, but SNAP users had higher rates of child uncooperativeness than non-SNAP users	Monetary support helps parental anger and irritability (expansion of Child Tax Credit cited)
Wang et al., (2020)	SNAP participation versus no SNAP participation	Parenting stress (i.e. the stress that comes from parenting) and parental engagement (e.g. time spent playing with children, reading to children, praising children, etc.)	4	SNAP reduced parental stress but also reduced parental engagement	None
Abuse or neglect of children or other family members					
Austin et al., (2023)	SNAP participation before versus after a policy/implementation change (i.e. natural experiment)	Total CPS reports, neglect-related CPS reports, and physical-abuse-related CPS reports	5	Both total and neglect-related CPS reports decreased after the elimination of the asset test and the adoption of policies that increased the income limit for SNAP separately. If a state enacted both policies, it led to a decrease in total, neglect-related, and physical-abuse-related CPS reports	Elimination of the state asset test and increasing the income limit for SNAP both decrease overall child maltreatment and several types of child maltreatment, especially child neglect, although physical abuse is harder to change through SNAP
Bullinger et al., (2021)	The distance between households and the SNAP authorized retailers-as a measure of ease or difficulty of using one’s SNAP benefits	Child maltreatment reports	5	There is no relationship between nearby SNAP stores and child maltreatment rates, with the exception of rural areas; in rural areas, proximity to a SNAP store decreases child maltreatment reports	The study suggests making it administratively easier for stores to accept SNAP, or to subsidize building new grocery stores; the authors also caution policymakers to consider the costs of child abuse when doing cost–benefit analyses of SNAP
Carr & Packham, (2020)	SNAP participation before versus after a policy/implementation change (i.e. natural experiment)	Domestic abuse and child-maltreatment	5	Shifting the benefit receipt date from the first of the month to a range of dates later in the month increased domestic abuse and child maltreatment, driven by an increase in crimes in the last three weeks of the month	Staggering receipt dates leads to a large short-run increase in domestic violence, and policy makers should be aware of this and its costs when weighing against the long-run decrease in theft
Cho & Lightfoot, (2022)	SNAP participation versus no SNAP participation	Risk for repeated child maltreatment substantiations	4	If a parent had disabilities, SNAP benefits increased the risk of repeated maltreatment substantions	There needs to be much more of a focus on the needs of disabled parents in further research and policy
Johnson-Motoyama et al., (2022)	Differences across State policy/implementation	Reports of child maltreatment accepted for CPS investigation, children in substantiated reports, and children receiving foster care services for all forms of maltreatment, and specifically for child neglect per 100 000 child population	5	Greater SNAP income generosity decreased CPS reports significantly	Calls for a policy focus on the stability of household resources as well as increasing SNAP benefits
Millett et al., (2011)	SNAP participation versus no SNAP participation	"Child maltreatment measures include aggregate numbers of screened in reports (six states), screened out reports (two states), total reports (three states), and reports by maltreatment type (three states)."	4	Food stamp usage usually reduced child maltreatment rates	None
Morris et al., (2019)	SNAP participation versus no SNAP participation	Substantiated abuse/neglect cases	5	A higher percentage of children receiving SNAP benefits was associated with a higher risk of substantiated child maltreatment over space and time, controlling for several factors (including socioeconomic)	None
Xu et al., (2021)	SNAP participation versus no SNAP participation	Child neglect	4	SNAP decreased the risk of child neglect, especially in poorer households	Signing up for and receiving SNAP has a lot of bureaucractic hurdles in place that make it hard for grandparents and foster parents to get help for their children; this study suggests altering requirements and processes to lessen these hurdles

### Impact on Child Development and Behavior

A slight majority of studies (n = 6) found that SNAP was correlated with improvements in child development and behavior, (Barr & Smith, 2022; Bolbocean & Tylavsky, 2021; East, 2020; Ettinger de Cuba et al., 2019a, 2019b; Hong & Henly, 2020), although results were mixed. Others (n = 5) found that SNAP participation and the timing of SNAP benefit receipt was negatively associated with child development and behavior (Cotti et al., 2018; Gassman-Pines & Bellows, 2018; Gennetian et al., 2016; Hong et al., 2020; Rothbart & Heflin, 2023). Some of these studies look at ecological data including data on poverty or the impact of SNAP policies (n = 2). Others study only welfare or specifically SNAP participants past or present (n = 6), and still others look at income or other poverty measures to control the effect of income on SNAP (n = 3). SNAP benefits had a net positive correlation with child development (Bolbocean & Tylavsky, 2021; East, 2020; Ettinger de Cuba et al., 2019a, 2019b), reduced the likelihood of later criminal conviction (Barr & Smith, 2022), and improved math skills among students in deep poverty (Hong & Henly, 2020). On the negative side, SNAP-participating students received lower scores on various tests than their non-SNAP peers (Rothbart & Heflin, 2023), performed worse on tests immediately before and after SNAP distribution (Cotti et al., 2018; Gassman-Pines & Bellows), and had a higher likelihood of bullying, being bullied, and general disciplinary infractions (Gennetian et al., 2016; Hong et al., 2020). For a full list of results, see Table 2.

### Mental Health

Participating in or increased SNAP benefits were largely associated with improved mental health in children and parents across studies (n = 7) (Bergmans et al., 2017; Ettinger de Cuba et al., 2019b; Frank & Sato, 2022; Munger et al., 2016; Pryor et al., 2023; Steimle et al., 2021; Wang et al., 2020). All studies listed either have a study population made up entirely of past or present SNAP participants (n = 3) and/or control for income level or other resource measures (n = 4). Two studies measured SNAP as secondary or moderating variables. SNAP participation was associated with reduced maternal depression (Munger et al., 2016), and loss of SNAP was associated with increased maternal depression (Ettinger de Cuba et al., 2019b; Munger et al., 2016). However, one article suggested that SNAP benefits only decreased maternal depression if the mother did not think SNAP or government-sponsored programs limited her personal freedom (Bergmans et al., 2017). SNAP participation was associated with decreased parental stress among most studies. Parental stress increased in one study because children in SNAP-participating families were less cooperative than children in non-SNAP families, presumably due to school closures affecting one group more than the other (Steimle et al., 2021). Another study found that while SNAP participation decreased parental stress, it also decreased parental engagement due to the requirements of food preparation crowding out spending time together (Wang et al., 2020). For adolescents, SNAP was found to buffer the harmful effects of food insecurity on depression and other mental health concerns later in young adulthood (Frank & Sato, 2022; Pryor et al., 2023).

### Abuse or Neglect

A majority of studies found positive impacts on abuse and neglect associated with SNAP participation (n = 8) (Austin et al., 2023; Bullinger et al., 2021; Carr & Packham, 2020; Cho & Lightfoot, 2022; Johnson-Motoyama et al., 2022; Millett et al., 2011; Morris et al., 2019; Xu et al., 2021). Some of these studies looked at SNAP as a secondary factor (n = 3). Others looked at the effects of SNAP policy changes (n = 3). Still others accounted for potential confounding variables in other ways (n = 2), such as looking at ecological data on income or unemployment. Various studies (n = 4) found that SNAP participation was associated with less child maltreatment (Austin et al., 2023; Johnson-Motoyama et al., 2022; Millett et al., 2011; Xu et al., 2021). However, Morris and colleagues (2019) found that counties with higher percentages of SNAP participation were associated with higher risk of child abuse and neglect, though these results could be due to self-selection bias. Similarly, Cho and Lightfoot (2022) observed that for parents with disabilities, receiving SNAP benefits significantly increased the risk for substantiated child maltreatment reports. Other articles indicated that child maltreatment decreases when monthly SNAP benefits are distributed at the beginning of the month rather than distributed throughout the month (Carr & Packham, 2020) and as proximity to retailers that accept SNAP benefit cards increases in rural areas (Bullinger et al., 2021).

## Discussion

**Fig. 2:**
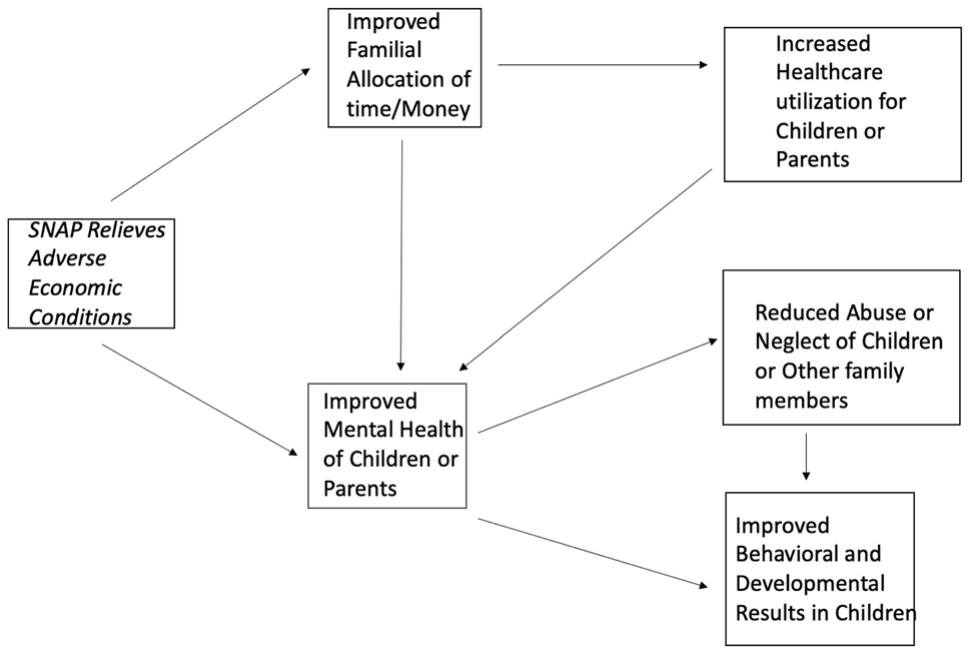
Proposed mechanism by which SNAP relieves adverse economic conditions and improves family outcomes

The Family Stress Model seeks to explain how stressors such as poverty create many adverse outcomes (Conger et al., 1994). In this model, as families encounter economic hardship, the interplay between economic pressure, parental depression, and marital conflict negatively impacts child socio-emotional development, school engagement, academic performance, and health outcomes. Poverty disrupts healthy family functioning and supportive parent–child relationships (Gard et al., 2020). Overall, SNAP participation was associated with clear improvements in four of the five categories of family outcomes, with one of the family outcomes having only a slim majority of positive results. Taking the Family Stress Model as a foundation, we propose Fig. 2 as a possible mechanism and ecology by which SNAP improves family outcomes. We do not have the data to definitively establish causation, making this a valuable area for future research.

### Family Healthcare Utilization

Overall, SNAP participation is associated with increased healthcare use. This positive connection between SNAP and health care has several potential causes. First, improving basic nutrition improves health, thus diminishing the need for medical treatments as a result of better food choices. Similarly, Serchen et al., 2022 found that leveraging nutrition assistance programs during the pandemic resulted in improved public health. Second, SNAP participation can reduce the likelihood of participants being forced to choose between paying for food or paying for medicine. For example, money saved through SNAP, though modest, may help offset a portion of current or future costs of medications (Serchen et al., 2022). Third, SNAP participants with chronic diseases—such as diabetes, hypertension, and coronary heart disease—are shown to experience improved cost savings compared to nonparticipants (Carlson & Keith-Jennings, 2018; Lee et al., 2019; Liu & Eicher-Miller, 2021). Our findings support conclusions that would be drawn from the Family Stress Model: that availability of SNAP resources increases family healthcare use.

Many studies explored the association between SNAP participation, health outcomes, and healthcare use (n = 9). Most of these studies mentioned “foregone healthcare visits.” Based on the number and commonality of dependent variables, we recommend a robust variance estimation meta-analysis to identify how SNAP affects healthcare access or costs—a key consideration for policymakers, healthcare providers, and other involved parties.

### Family Mental Health

Several studies found that SNAP participation was strongly associated with improved mental health in children and parents. These findings support research that associates decreased parental stress with decreased child stress through biological pathways (Lupien et al., 2000; Yoshikawa et al., 2012). Better nutrition through SNAP may also improve mental health (Grajek et al., 2022). These strong associations between SNAP participation and improved mental health outcomes validate the Family Stress Model and show potential mechanisms by which SNAP improves mental health for the whole family.

### Familial Allocation of Resources

Many studies found a correlation between SNAP participation and improved family resource allocation. SNAP enabled households to devote more money to non-food-related payments than non-SNAP-participating households (Kim, 2016). However, SNAP families still faced difficulties, especially near the end of the month. Coping mechanisms included borrowing money, parents skipping meals to feed children, and the whole family eating more unhealthy food. Difficulties may arise from a variety of factors, including benefit amounts being too low to last throughout the month (Nieves et al., 2022; Schenck-Fontaine et al., 2017), lack of financial literacy (Weinstein et al., 2018), and SNAP distribution occurring only once a month (Schenck-Fontaine et al., 2017).

### Child Behavior and Development

In general, SNAP participation was associated with improved behavioral and developmental outcomes in children. The SNAP cycle is a recurring theme—often, positive behavioral outcomes decrease towards the end of the SNAP month. Our results suggest that the positive economic effects of SNAP benefits decrease as the month goes on because of resources running low, which findings align with the Family Stress Model. Research shows that child behavior is vulnerable to slight shifts in nutrition (Shankar et al., 2017), which may occur as families employ coping strategies that shift to less nutritious but more affordable foods consumed towards the end of the benefit cycle. Additionally, extreme financial hardship is considered an adverse childhood experience (ACE). ACEs in early childhood are linked to behavioral problems in children as they grow up (Choi et al., 2019).

### Abuse and Neglect

A majority of studies found improved abuse and neglect markers associated with SNAP. Limited access to approved SNAP retail stores, parental disability, inefficient timing of benefits, and strict income eligibility were major reasons for the results found on SNAP participation and familial abuse and neglect. Nevertheless, the majority of studies have shown that SNAP eligibility is associated with fewer cases of abuse and neglect. A recent study by Austin et al. (2023) on expanding SNAP income eligibility by eliminating the asset test—currently an optional policy action at the state level—resulted in significant reductions in CPS investigations of child maltreatment. This finding increased over time for Black and white children, affirming the positive impact of family-friendly policies on child health and well-being. Policy changes that increase SNAP accessibility address both food insecurity and child maltreatment which are among several major ACEs (Bethell et al., 2017). Helping families become food-secure can improve family functioning through effective resource allocation, which in turn promotes healthy interactions within the family.

### Policy Actions Suggested

Two primary policy actions emerged from our work, which are directed toward state agencies managing SNAP benefits, state and federal policymakers legislating SNAP, and food retailers selling SNAP food items. These actions may enhance SNAP’s ability to nourish needy families, stabilize household resources, improve healthcare usage, and reduce poverty among needy households (Carlson & Keith-Jennings, 2018). Equally important, these policy actions, including reducing some of the administrative barriers noted above, may help address some unintentional and unanticipated adverse consequences of SNAP participation. These are achievable priorities, given a reasonably steady history of bipartisan political support for many essential policy improvements to SNAP, including more recent discussions formalized in 2018 and 2020 (Franckle et al., 2019).

First, distributing SNAP benefits throughout the month rather than once a month is a leading policy change priority. While benefit amounts and eligibility may remain the same, distributing SNAP resources at least twice a month may help families stretch SNAP benefits more effectively and provide many benefits, including improved diet quality, better healthcare usage, mental health, better budgeting, and other positive benefits (Cotti, 2020; Bronchetti et al., 2019).

Second, eliminate an eligibility benefit cliff by gradually reducing benefits when income limits and work requirements deadlines pass for families with children. Softening the abrupt end of SNAP participation when wages increase or work requirement deadlines pass is a frequently mentioned policy change priority (Ettinger de Cuba, 2019b; Hoynes & Schanzenbach, 2012; Neuert et al., 2019; Karpman et al., 2019; Gassman-Pines & Schenck‐Fontaine, 2019).

As identified by other studies, future research should focus on additional policy actions for SNAP since they build from or complement the policy actions we identified. First, increasing SNAP benefit payments to match the cost of food, especially with current inflationary trends, is critical to research. For example, research could consider how regionally adjusted benefits to local food prices may help policymakers consider how much SNAP benefits may change (Austin et al., 2023; Christensen & Bronchetti, 2020; Gregory & Coleman-Jensen, 2013). Second, coordinating SNAP eligibility and enrollment with other welfare services is recommended by studies outside this scoping review (Herman et al., 2023; Serchen et al., 2022; Thorndike et al., 2022). Researchers should study how and why better coordination for welfare system enrollments may reduce or end some cyclical issues that SNAP participants face.

Future research on the family health impact of SNAP needs better assessment measures. The Family Health Scale (FHS) (Crandall et al., 2020), in short or long forms, and the Family Star Plus assessment (Good & MacKeith, 2021) can help evaluate the multiple dimensions of family impact and the availability and implementation of family-friendly policies. Measures of policy and health impact on the family as a collective unit (Prime et al., 2020) may also be employed.

### Limitations

Scoping reviews have a broader focus than systematic reviews. With the emphasis on breadth, there was the possibility of missing critical details that aid in interpreting study findings. We attempted to provide depth by considering studies that included dependent variables related to family impact measures. Additionally, of the 46 studies in this scoping review, only two were qualitative in nature with one mixed-methods study, thus potentially limiting the integration of richer perspectives from a broader range of study designs. While this limitation was beyond our control, it reflected the limited use of qualitative and mixed methods studies on the family implications of SNAP participation. Further, 58% of the articles passed all 5 methodological quality criteria, and an additional 34% satisfied 4 criteria, potentially reflecting a need for deeper empirical methodology in studies addressing family outcomes. Understanding the full impact of SNAP could be gained by employing a broader range of study methodologies.

## Conclusion

SNAP is a highly effective program with growing evidence that it positively impacts family health and alleviates poverty. While more research needs to be conducted on the non-food-related impacts of SNAP on family health and poverty, two policy actions resulting from this scoping review deserve attention from policymakers, program administrators, and retail food establishments.

## Data Availability

Available upon request.

## References

[CR1] Almada L, McCarthy IM (2017). It’s a cruel summer: Household responses to reductions in government nutrition assistance. Journal of Economic Behavior & Organization.

[CR2] Andreyeva T, Tripp AS, Schwartz MB (2015). Dietary quality of Americans by supplemental nutrition assistance program participation status: A systematic review. American Journal of Preventive Medicine.

[CR3] Arteaga I, Hodges L, Heflin C (2021). Giving kids a boost: The positive relationship between frequency of SNAP participation and Infant’s preventative health care utilization. SSM - Population Health.

[CR4] Austin AE, Shanahan ME, Frank M, Naumann RB, McNaughton Reyes HL, Corbie G, Ammerman AS (2023). Association of state expansion of supplemental nutrition assistance program eligibility with rates of child protective services–investigated reports. Archives of Pediatrics & Adolescent Medicine.

[CR5] Barr A, Smith AA (2022). Fighting crime in the cradle: The effects of early childhood access to nutritional assistance. The Journal of Human Resources.

[CR6] Beatty TK, Nanney MS, Tuttle C (2013). Time to eat? The relationship between food security and food-related time use. Public Health Nutrition.

[CR7] Bensignor MO, Freese RL, Sherwood NE, Berge JM, Kunin-Batson A, Veblen-Mortenson S, French SA (2021). The relationship between participation, parent feeding styles, and child eating behaviors. Journal of Hunger & Environmental Nutrition.

[CR8] Bergmans RS, Berger LM, Palta M, Robert SA, Ehrenthal DB, Malecki K (2017). Participation in the supplemental nutrition assistance program and maternal depressive symptoms: Moderation by program perception. Social Science & Medicine.

[CR9] Bethell CD, Carle A, Hudziak J, Gombojav N, Powers K, Wade R, Braveman P (2017). Methods to assess adverse childhood experiences of children and families: Toward approaches to promote child well-being in policy and practice. Academic Pediatrics.

[CR10] Bolbocean C, Tylavsky FA (2021). The impact of safety net programs on early-life developmental outcomes. Food Policy.

[CR11] Breck, A. (2018). *Effect of the Supplemental Nutrition Assistance Program on Health and Healthcare Expenditures*. Available from Dissertation Abstracts International http://www.pqdtcn.com/thesisDetails/2D2BB2F062A61CAFB7033265C9E5FD18

[CR12] Bronchetti ET, Christensen G, Hoynes HW (2019). Local food prices, SNAP purchasing power, and child health. Journal of Health Economics.

[CR13] Bullinger LR, Fleckman JM, Fong K (2021). Proximity to SNAP-authorized retailers and child maltreatment reports. Economics and Human Biology.

[CR14] Carlson, S., & Keith-Jennings, B. (2018). *SNAP is linked with improved nutritional outcomes and lower healthcare costs.* Targeted News Service. https://search.proquest.com/docview/1988493113

[CR15] Carr JB, Packham A (2020). SNAP schedules and domestic violence. Journal of Policy Analysis and Management.

[CR16] Caswell JA, Yaktine AL, Committee on Examination of the Adequacy of Food Resources and SNAP Allotments, Food and Nutrition Board, Committee on National Statistics, Institute of Medicine, & National Research Council (2013). Supplemental Nutrition Assistance Program: Examining the Evidence to Define Benefit Adequacy.

[CR17] Cellini SR, McKernan S, Ratcliffe C (2008). The dynamics of poverty in the United States: A review of data, methods, and findings. Journal of Policy Analysis and Management.

[CR18] Center on Budget and Policy Priorities. (2023). *A Closer Look at Who Benefits from SNAP: State-by-State Fact Sheets* [Fact sheet]. https://www.cbpp.org/research/food-assistance/a-closer-look-at-who-benefits-from-snap-state-by-state-fact-sheets#Alabama

[CR19] Cheng T (2010). Financial self-sufficiency or return to welfare? A longitudinal study of mothers among the working poor. International Journal of Social Welfare.

[CR20] Cho M, Lightfoot E (2022). Recurrence of substantiated maltreatment reports between low-income parents with disabilities and their propensity-score matched sample without disabilities. Child Maltreatment.

[CR21] Choi J, Wang D, Jackson AP (2019). Adverse experiences in early childhood and their longitudinal impact on later behavioral problems of children living in poverty. Child Abuse & Neglect.

[CR22] Christensen G, Bronchetti ET (2020). Local food prices and the purchasing power of SNAP benefits. Food Policy.

[CR23] Collyer S, Gandhi J, Garfinkel I, Ross S, Waldfogel J, Wimer C (2022). The effects of the 2021 monthly child tax credit on child and family well-being: Evidence from New York City. Socius: Sociological Research for a Dynamic World.

[CR24] Conger RD, Ge X, Elder GH, Lorenz FO, Simons RL (1994). Economic stress, coercive family process, and developmental problems of adolescents. Child Development.

[CR25] Cotti CD, Gordanier JM, Ozturk OD (2020). Hunger pains? SNAP timing and emergency room visits. Journal of Health Economics.

[CR26] Cotti C, Gordanier J, Ozturk O (2018). When does it count? The timing of food stamp receipt and educational performance. Economics of Education Review.

[CR27] Crandall A, Weiss-Laxer NS, Broadbent E, Holmes EK, Magnusson BM, Okano L, Berge JM, Barnes MD, Hanson CL, Jones BL, Novilla LB (2020). The family health scale: Reliability and validity of a short- and long-form. Frontiers in Public Health.

[CR28] East C (2020). The effect of food stamps on children’s health: Evidence from immigrants’ hanging eligibility. The Journal of Human Resources.

[CR29] Engel K, Ruder EH (2020). Fruit and vegetable incentive programs for supplemental nutrition assistance program (SNAP) participants: A scoping review of program structure. Nutrients.

[CR30] Engle PL, Black MM (2008). The effect of poverty on child development and educational outcomes. Annals of the New York Academy of Sciences.

[CR31] Ettinger de Cuba SA, Bovell-Ammon AR, Cook JT, Coleman SM, Black MM, Chilton MM, Casey PH, Cutts DB, Heeren TC, Sandel MT, Sheward R, Frank DA (2019). SNAP, young children’s health, and family food security and healthcare access. American Journal of Preventive Medicine.

[CR32] Ettinger de Cuba S, Chilton M, Bovell-Ammon A, Knowles M, Coleman SM, Black MM, Cook JT, Cutts DB, Casey PH, Heeren TC, Frank DA (2019). Loss of SNAP is associated with food insecurity and poor health in working families with young children. Health Affairs.

[CR33] Franckle RL, Polacsek M, Bleich SN, Thorndike AN, Findling MTG, Moran AJ, Rimm EB (2019). Support for supplemental nutrition assistance program (SNAP) policy alternatives among US adults, 2018. American Journal of Public Health (1971).

[CR34] Frank ML, Sato AF (2022). Food insecurity and depressive symptoms among adolescents: Does federal nutrition assistance act as a buffer?. Journal of Developmental and Behavioral Pediatrics.

[CR35] Gard AM, McLoyd VC, Mitchell C, Hyde LW (2020). Evaluation of a longitudinal family stress model in a population-based cohort. Social Development (oxford, England).

[CR36] Gassman-Pines A, Schenck-Fontaine A (2019). Daily food insufficiency and worry among economically disadvantaged families with young children. Journal of Marriage and Family.

[CR37] Gassman-Pines A, Bellows L (2018). Food instability and academic achievement: A quasi-experiment using SNAP benefit timing. American Educational Research Journal.

[CR38] Gennetian LA, Seshadri R, Hess ND (2016). Supplemental nutrition assistance program (SNAP) benefit cycles and student disciplinary. Social Service Review.

[CR39] Good A, MacKeith J (2021). Assessing family functioning: Psychometric evaluation of the family Star Plus. Family Relations.

[CR40] Grajek M, Krupa-Kotara K, Białek-Dratwa A, Sobczyk K, Grot M, Kowalski O, Staśkiewicz W (2022). Nutrition and mental health: A review of current knowledge about the impact of diet on mental health. Frontiers in Nutrition (lausanne).

[CR41] Gregory CA, Coleman-Jensen A (2013). Do high food prices increase food insecurity in the United States?. Applied Economic Perspectives and Policy.

[CR42] Gupta RP, de Wit ML, McKeown D (2007). The impact of poverty on the current and future health status of children. Paediatrics & Child Health.

[CR43] Heflin C, Hodges L, Mueser P (2017). Supplemental nutrition assistance program benefits and emergency room visits for hypoglycaemia. Public Health Nutrition.

[CR44] Herman WH, Schillinger D, Bolen S, Boltri JM, Bullock A, Chong W, Conlin PR, Cook JW, Dokun A, Fukagawa N, Gonzalvo J, Greenlee MC, Hawkins M, Idzik S, Leake E, Linder B, Lopata AM, Schumacher P, Shell D, Wu S (2023). The National Clinical Care Commission report to Congress: recommendations to better leverage federal policies and programs to prevent and control diabetes. Diabetes Care.

[CR45] Holley CE, Mason C (2019). A systematic review of the evaluation of interventions to tackle children’s food insecurity. Current Nutrition Reports.

[CR46] Hong JS, Choi J, Espelage DL, Wu C, Boraggina-Ballard L, Fisher BW (2020). Are children of welfare recipients at a heightened risk of bullying and peer victimization?. Child & Youth Care Forum.

[CR47] Hong QN, Fàbregues S, Bartlett G, Boardman F, Cargo M, Dagenais P, Gagnon M, Griffiths F, Nicolau B, O’Cathain A, Rousseau M, Vedel I, Pluye P (2018). The mixed methods appraisal tool (MMAT) version 2018 for information professionals and researchers. Education for Information.

[CR48] Hong YS, Henly JR (2020). Supplemental nutrition assistance program and school readiness skills. Children and Youth Services Review.

[CR49] Hoynes HW, Schanzenbach DW (2012). Work incentives and the food stamp program. Journal of Public Economics.

[CR50] Hoynes HW, Schanzenbach DW, Almond D (2016). Long-run impacts of childhood access to the safety net. The American Economic Review.

[CR51] Hudak KM, Racine EF (2019). The supplemental nutrition assistance program and child weight status: A review. American Journal of Preventive Medicine.

[CR52] Johnson-Motoyama M, Ginther DK, Oslund P, Jorgenson L, Chung Y, Phillips R, Beer OWJ, Davis S, Sattler PL (2022). Association between state supplemental nutrition assistance program policies, child protective services involvement, and foster care in the US, 2004–2016. JAMA Network Open.

[CR53] Karpman, M., Hahn, H., & Gangopadhyaya, A. (2019). *Precarious work schedules could jeopardize access to safety net programs targeted by work requirements.*Washington, DC: Urban Institute. https://search.proquest.com/docview/2250577999

[CR54] Kim J (2016). Do SNAP participants expand non-food spending when they receive more SNAP benefits?—Evidence from the 2009 SNAP benefits increase. Food Policy.

[CR55] Kinsey EW, Oberle M, Dupuis R, Cannuscio CC, Hillier A (2019). Food and financial coping strategies during the monthly supplemental nutrition assistance program cycle. SSM - Population Health.

[CR56] Laurito A, Schwartz AE (2019). Does school lunch fill the “SNAP Gap” at the end of the month?. Southern Economic Journal.

[CR57] Lee K, Zhang L (2022). Cumulative effects of poverty on children’s social-emotional development: Absolute poverty and relative poverty. Community Mental Health Journal.

[CR58] Lee Y, Mozaffarian D, Sy S, Huang Y, Liu J, Wilde PE, Abrahams-Gessel S, Jardim TdSV, Gaziano TA, Micha R (2019). Cost-effectiveness of financial incentives for improving diet and health through medicare and medicaid: A microsimulation study. PLoS Medicine.

[CR59] Liu Y, Eicher-Miller HA (2021). Food insecurity and cardiovascular disease risk. Current Atherosclerosis Reports.

[CR60] Lupien SJ, King S, Meaney MJ, McEwen BS (2000). Child’s stress hormone levels correlate with mother’s socioeconomic status and depressive state. Biological Psychiatry.

[CR61] Mabli J, Worthington J (2017). Supplemental Nutrition Assistance Program participation and emergency food pantry use. Journal of Nutrition Education and Behavior.

[CR62] Maguire-Jack K, Johnson-Motoyama M, Parmenter S (2022). A scoping review of economic supports for working parents: The relationship of TANF, child care subsidy, SNAP, and EITC to child maltreatment. Aggression and Violent Behavior.

[CR63] Mande J, Flaherty G (2023). Supplemental Nutrition Assistance Program as a health intervention. Current Opinion in Pediatrics.

[CR64] McKernan S, Ratcliffe C, Braga B (2021). The effect of the US safety net on material hardship over two decades. Journal of Public Economics.

[CR65] Miller DP, Morrissey TW (2021). SNAP participation and the health and health care utilisation of low-income adults and children. Public Health Nutrition.

[CR66] Millett L, Lanier P, Drake B (2011). Are economic trends associated with child maltreatment? Preliminary results from the recent recession using state level data. Children and Youth Services Review.

[CR67] Morris MC, Marco M, Maguire-Jack K, Kouros CD, Im W, White C, Bailey B, Rao U, Garber J (2019). County-level socioeconomic and crime risk factors for substantiated child abuse and neglect. Child Abuse & Neglect.

[CR68] Morrissey TW, Miller DP (2020). Supplemental nutrition assistance program participation improves children’s health care cse: An analysis of the American Recovery and Reinvestment Act’s natural experiment. Academic Pediatrics.

[CR69] Munger AL, Hofferth SL, Grutzmacher SK (2016). The role of the supplemental nutrition assistance program in the relationship between food insecurity and probability of maternal depression. Journal of Hunger & Environmental Nutrition.

[CR70] Neuert, H., Fischer, E., Darling, M., & Barrows, A. (2019). *Work Requirements Don’t Work: A Behavioral Science Perspective.* Ideas, 42. http://www.ideas42.org/wp-content/uploads/2019/04/ideas42-Work-Requirements-Paper.pdf

[CR71] Nieves C, Dannefer R, Zamula A, Sacks R, Ballesteros Gonzalez D, Zhao F (2022). “Come with us for a week, for a month, and see how much food lasts for you:” A Qualitative Exploration of Food Insecurity in East Harlem, New York City. Journal of the Academy of Nutrition and Dietetics.

[CR72] Page MJ, McKenzie JE, Bossuyt PM, Boutron I, Hoffmann TC, Mulrow CD, Shamseer L, Tetzlaff JM, Akl EA, Brennan SE, Chou R, Glanville J, Grimshaw JM, Hróbjartsson A, Lalu MM, Li T, Loder EW, Mayo-Wilson E, McDonald S, Moher D (2021). The PRISMA 2020 statement: An updated guideline for reporting systematic reviews. Journal of Clinical Epidemiology.

[CR73] Parolin Z (2021). Income support policies and the rise of student and family homelessness. The Annals of the American Academy of Political and Social Science.

[CR74] Powell TW, Wallace M, Zelaya C, Davey-Rothwell MA, Knowlton AR, Latkin CA (2018). Predicting household residency among youth from vulnerable families. Children and Youth Services Review.

[CR75] Prime H, Wade M, Browne DT (2020). Risk and resilience in family well-being during the COVID-19 pandemic. American Psychologist.

[CR76] Pryor L, Melchior M, Avendano M, Surkan PJ (2023). Childhood food insecurity, mental distress in young adulthood and the supplemental nutrition assistance program. Preventive Medicine.

[CR77] Rogers S, Garg A, Tripodis Y, Brochier A, Messmer E, Gordon Wexler M, Peltz A (2022). Supplemental nutrition assistance program participation and health care expenditures in children. BMC Pediatrics.

[CR78] Rothbart MW, Heflin C (2023). Inequality in literacy skills at kindergarten entry at the intersections of social programs and race. Children and Youth Services Review.

[CR79] Ryan-Ibarra, S., DeLisio, A., Bang, H., Adedokun, O., Bhargava, V., Franck, K., Funderburk, K., Lee, J. S., Parmer, S., & Sneed, C. (2020). The US supplemental nutrition assistance program—education improves nutrition-related behaviors. *Journal of Nutritional Science (Cambridge), 9*, 44. 10.1017/jns.2020.37 10.1017/jns.2020.37 PMC7731638 33343892

[CR80] Schenck-Fontaine A, Gassman-Pines A, Hill Z (2017). Use of informal safety nets during the supplemental nutrition assistance program benefit cycle: How poor families cope with within-month economic instability. Social Service Review.

[CR81] Serchen J, Atiq O, Hilden D (2022). Strengthening food and nutrition security to promote public health in the United States: A position paper from the American College of Physicians. Annals of Internal Medicine.

[CR82] Shaefer HL, Gutierrez IA (2013). The supplemental nutrition assistance program and material hardships among low-income households with children. The Social Service Review (chicago).

[CR83] Shankar P, Chung R, Frank DA (2017). Association of food insecurity with children’s behavioral, emotional, and academic outcomes: A systematic review. Journal of Developmental and Behavioral Pediatrics : JDBP.

[CR84] Sonik RA (2016). Massachusetts inpatient medicaid cost response to increased supplemental nutrition assistance program benefits. American Journal of Public Health (1971).

[CR85] Steimle S, Gassman-Pines A, Johnson AD, Hines CT, Ryan RM (2021). Understanding patterns of food insecurity and family well-being amid the COVID-19 pandemic using daily surveys. Child Development.

[CR86] Thorndike AN, Gardner CD, Kendrick KB, Seligman HK, Yaroch AL, Gomes AV, Ivy KN, Scarmo S, Cotwright CJ, Schwartz MB (2022). Strengthening US food policies and programs to promote equity in nutrition security: A policy statement from the American Heart Association. Circulation.

[CR87] Vartanian TP, Houser L, Harkness J (2011). Food stamps and dependency: disentangling the short-term and long-term economic effects of food stamp receipt and low income for young mothers. Journal of Sociology and Social Welfare.

[CR88] Verghese A, Raber M, Sharma S (2019). Interventions targeting diet quality of supplemental nutrition assistance program (SNAP) participants: A scoping review. Preventive Medicine.

[CR89] Wagmiller RL, Adelman RM (2009). Childhood and intergenerational poverty: the long-term consequences of growing up poor. National Center for Children in Poverty.

[CR90] Wang JS, Zhao X, Nam J (2020). The effects of welfare participation on parenting stress and parental engagement using an instrumental variables approach: Evidence from the supplemental nutrition assistance program. Children and Youth Services Review.

[CR91] Weinstein O, Cordeiro LS, Ronnenberg A, Sartori A, Anderson ALW, Nelson-Peterman J (2018). What works when it comes to having enough: A qualitative analysis of SNAP-participants’ food acquisition strategies. Journal of Hunger & Environmental Nutrition.

[CR92] Xu Y, Jedwab M, Soto-Ramírez N, Levkoff SE, Wu Q (2021). Material hardship and child neglect risk amidst COVID-19 in grandparent-headed kinship families: The role of financial assistance. Child Abuse & Neglect.

[CR93] Yoshikawa H, Aber JL, Beardslee WR (2012). The effects of poverty on the mental, emotional, and behavioral health of children and youth. The American Psychologist.

